# Efficacy and Safety of Sofosbuvir-Velpatasvir combination in Hepatitis C Virus-infected Pakistani Patients without Cirrhosis or with Compensated Cirrhosis: A Prospective, Open-label Interventional Trial

**DOI:** 10.7759/cureus.6537

**Published:** 2020-01-01

**Authors:** Nazish Butt, Iltaf Muhammad, Attique Abou Bakr, Zaheer Akhtar, Mashhood Ali, Sharib Syed Muhammad, Neeta Maheshwary

**Affiliations:** 1 Gastroenterology, Jinnah Postgraduate Medical Centre, Karachi, PAK; 2 Gastroenterology, Hayatabad Medical Complex, Peshawar, PAK; 3 Gastroenterology, Jinnah Hospital, Lahore, PAK; 4 Gastroenterology, Gulab Devi Hospital, Lahore, PAK; 5 Gastroenterology, Pakistan Institute of Medical Sciences, Islamabad, PAK; 6 Clinical Research, Hilton Pharma (Pvt.) Ltd., Karachi, PAK

**Keywords:** hepatitis c, liver cirrhosis, sofosbuvir-velpatasvir, sustained virologic response, pakistan

## Abstract

Background

In Pakistan, there is a paucity of published clinical data regarding the efficacy of sofosbuvir-velpatasvir in the management of patients with hepatitis C without cirrhosis or with compensated cirrhosis.

Methods

A prospective, open-label, multicenter, interventional trial was conducted in patients with hepatitis C without cirrhosis or with compensated cirrhosis. Hepatitis C patients without cirrhosis or with compensated cirrhosis were screened, and 133 patients were enrolled in the study. They received sofosbuvir 400 mg plus velpatasvir 100 mg combination once daily for 12 weeks. Patients were followed up for six months after the start of therapy. Hepatitis C viral load was assessed at baseline, at week 12, and after 24 weeks following the start of the treatment. The trial was prospectively registered with the Iranian Registry of Clinical Trials (IRCT) with the identification number IRCT20170614034526N4.

Results

Among enrolled patients, 79 were male, and 54 were female. Ninety-five (71.4%) patients were without cirrhosis, and 38 had compensated cirrhosis. Patients without cirrhosis had a mean age of 45.90 ±10.99 years, and patients with compensated cirrhosis had a mean age of 52.60 ±12.29 years. As per the intention-to-treat analysis, all patients without cirrhosis and 35 (92.1%) patients with compensated cirrhosis achieved undetectable viral load hepatitis C virus (HCV) ribonucleic acid (RNA) of <15 IU/mL at 12 weeks from the start of treatment. Eighty-six (90.5%) patients without cirrhosis achieved sustained virologic response 12 weeks after the end of therapy. Patients with compensated cirrhosis experienced more adverse events (31.5%) than patients without cirrhosis (20.15%).

Conclusion

Direct-acting antiviral therapy using sofosbuvir and velpatasvir combination is effective and safe in HCV patients without cirrhosis and patients with compensated cirrhosis.

## Introduction

Chronic hepatitis C virus (HCV) infections have become a common global health problem, with an estimated 71 million people having been affected globally [1]. Pakistan is the second-largest HCV-prevalent country, with an estimated adult HCV seroprevalence of 4.5-8.2% [2,3]. Chronic HCV can lead to cirrhosis, decompensated liver disease, and hepatocellular carcinoma in 30-50% of infected patients [4]. In 2016, 399,000 people are estimated to have died from hepatitis C globally, mostly from cirrhosis and hepatocellular carcinoma [1]. Various drug therapies have been in use since 1991, such as conventional interferon and pegylated interferon plus ribavirin, and sustained virologic response (SVR) has been achieved in 40-45% in genotype 1, 80% in genotype 2, and 50% in genotype 3a patients. However, 50% to 60% of patients have also been reported as not responding to these therapies or relapsing after the initial recovery [5]. Interferon-related side effects include bone marrow depression, flu-like symptoms, neuropsychiatric disorders, and autoimmune syndromes, while the main problem with ribavirin is hemolytic anemia [6]. Limited SVR profile and associated side effects of these therapies have led the researchers to develop new safe and effective treatment options.

In the past decade, newer oral treatment regimens that act on nonstructural proteins have been introduced [7]. The use of direct-acting antiviral agents has resulted in cure rates of >90% regardless of liver fibrosis, previous treatment, or any demographic aspect [8]. Sofosbuvir has been the first nucleotide analog that provided better achievement of SVR on its own [9]. A twelve-week treatment regimen comprising sofosbuvir plus ribavirin with and without pegylated interferon has resulted in a substantial decrease in the viral ribonucleic acid (RNA) in HCV-infected patients of genotype 2 or 3 [10].

In June 2016, the US Food and Drug Administration (FDA) approved the sofosbuvir-velpatasvir combination for the treatment of adult patients with chronic HCV, both with and without cirrhosis. The sofosbuvir-velpatasvir combination is an oral treatment option for patients with chronic HCV of all six genotypes (i.e., genotypes 1 to 6) [11]. Sofosbuvir inhibits the hepatitis C NS5B protein [12]. Velpatasvir is an NS5A inhibitor, which is used together with sofosbuvir in the treatment of hepatitis C infection of all six major genotypes [13]. As per the European Association for Study of Liver Disease (EASL) 2018 guidelines, the sofosbuvir-velpatasvir combination is recommended in treatment-naïve or treatment-experienced hepatitis C patients without cirrhosis or with compensated cirrhosis for pan-genotypic infections [14].

In Pakistan, the sofosbuvir-velpatasvir combination has been approved for use since March 2018. This trial would be the first prospective interventional trial in Pakistan to focus on determining the efficacy and safety of the combination for the treatment of hepatitis C in patients without cirrhosis and patients with compensated cirrhosis in the local population.

## Materials and methods

Study design

A prospective, open-label, single-arm, multicenter interventional trial was conducted in the gastroenterology departments of various hospitals/medical colleges of Pakistan. The study followed the Consolidated Standards of Reporting Trials (CONSORT) guidelines to report the results of the trial.

Setting and participants

One hundred thirty-three patients with hepatitis C without cirrhosis and with compensated cirrhosis were enrolled in the gastroenterology departments of the following hospitals/medical colleges of Pakistan: Jinnah Postgraduate Medical Center in Karachi, the Hayatabad Medical Complex in Peshawar, the Gulab Devi Hospital in Lahore, Jinnah Hospital in Lahore, and the Pakistan Institute of Medical Sciences in Islamabad. All patients were enrolled using a nonprobability convenient sampling procedure. The study duration was from June 2018 to October 2019. Ethics committee approval was obtained from Jinnah Postgraduate Medical Center (F.2-81-IRB/2018-GENL/7065/JPMC). The study was also registered in the World Health Organization (WHO) clinical trial registry through the Iranian Registry of Clinical Trials (IRCT) with the identification number IRCT20170614034526N4.

Study process and interventions

**Figure 1:**
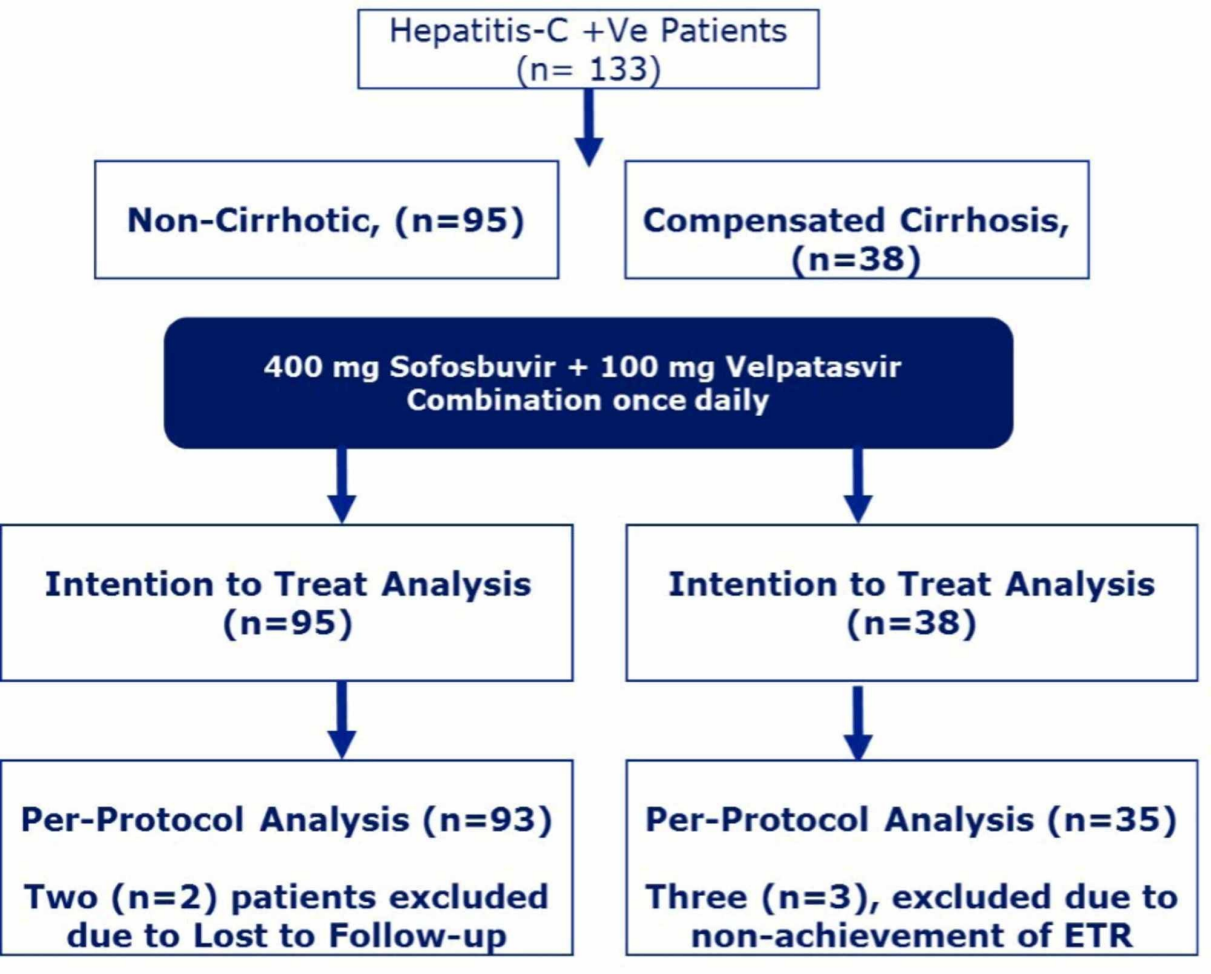
Patient distribution ETR: end-of-treatment response

All hepatitis C patients without cirrhosis or with compensated cirrhosis with detectable HCV RNA by polymerase chain reaction (PCR) meeting eligibility criteria were included. Patients having co-infection with hepatitis B virus (HBV) or HIV and terminally ill patients were excluded from the study (Figure 1).

Written informed consent was obtained from all patients. After baseline clinical examination and confirmation of HCV infection, patients were prescribed Hilvel® (400 mg sofosbuvir plus velpatasvir 100 mg combination; Hilton Pharma (Pvt) Ltd., Karachi, Pakistan) once daily for 12 weeks. Demographic variables and baseline investigations, including complete blood count, liver function tests, and detectable HCV load were noted.

Patients were classified as not having cirrhosis or having compensated cirrhosis based on clinical data and the Child-Pugh score. Both patients without cirrhosis and with compensated cirrhosis received the once-daily sofosbuvir-velpatasvir combination for a 12-week period. Two patients without cirrhosis and three patients with compensated cirrhosis were lost to follow-up after 12 weeks of treatment. Study results are reported for both intention-to-treat and per-protocol populations.

Outcomes

HCV viral load by Abbott RealTime (Abbott Laboratories, Abbott Park, IL) HCV PCR assay with a detection limit of <15 IU/mL was obtained at week 12 and week 24. The primary endpoint was the achievement of SVR. An SVR was defined as an undetectable viral load at 24 weeks from the start of therapy. The secondary outcome was the achievement of the end-of-treatment response (ETR) 12 weeks after the start of treatment.

Statistical analysis

All data were entered and analyzed on IBM SPSS Statistics for Windows, Version 21.0 (IBM Corp., Armonk, NY). Frequencies and percentages were measured for the qualitative variables. Mean and standard deviations were reported for quantitative data. Two-sided 95% exact conﬁdence intervals (CIs) for SVR24 based on the Clopper-Pearson method were provided for both patients without cirrhosis and patients with compensated cirrhosis. Primary outcomes (SVR and ETR) are analyzed and reported for intention-to-treat and per-protocol populations.

## Results

Among the 133 enrolled patients, 59.5% were male and 40.5% were female. Among them, 95 (71.4%) were without cirrhosis and 38 (28%) had compensated cirrhosis. Patients without cirrhosis had a mean age of 45.90 ±10.99 years, while patients with compensated cirrhosis had a mean age of 52.60 ±12.29 years. Child-Turcotte-Pugh (CTP) class, CTP score, and other baseline characteristics are presented in Table 1.

**Table 1:** Baseline characteristics in patients without cirrhosis and in patients with compensated cirrhosis CTP: Child-Turcotte-Pugh.

Variables (n = 133)		Patients without cirrhosis (n = 95)	Patients with compensated cirrhosis (n = 38)
		Mean ±SD (%)	Mean ±SD (%)
Age (years)		45.90 ±10.99	52.60 ±12.29
Gender	Male	54 (57)	25 (66)
	Female	41 (43)	13 (34)
CTP class A patients		___	38
CTP score		___	5.87 ±0.52
Model for end-stage liver disease score		___	7.84 ±1.07
Treatment experienced		7 (7.3)	0
Total bilirubin (mg/dL)		0.67 ±0.17	0.86 ±0.17
Alanine aminotransferase (U/L)		56.01 ±33.24	53.84 ±9.38
Serum creatinine (mg/dL)		0.76 ±0.24	0.77 ±0.19
Hemoglobin (g/dl)		12.82 ±1.35	11.16 ±1.25
Platelets x10^3^/mm^3^		254.31 ±65.34	112.78 ±20.01

**Figure 2:**
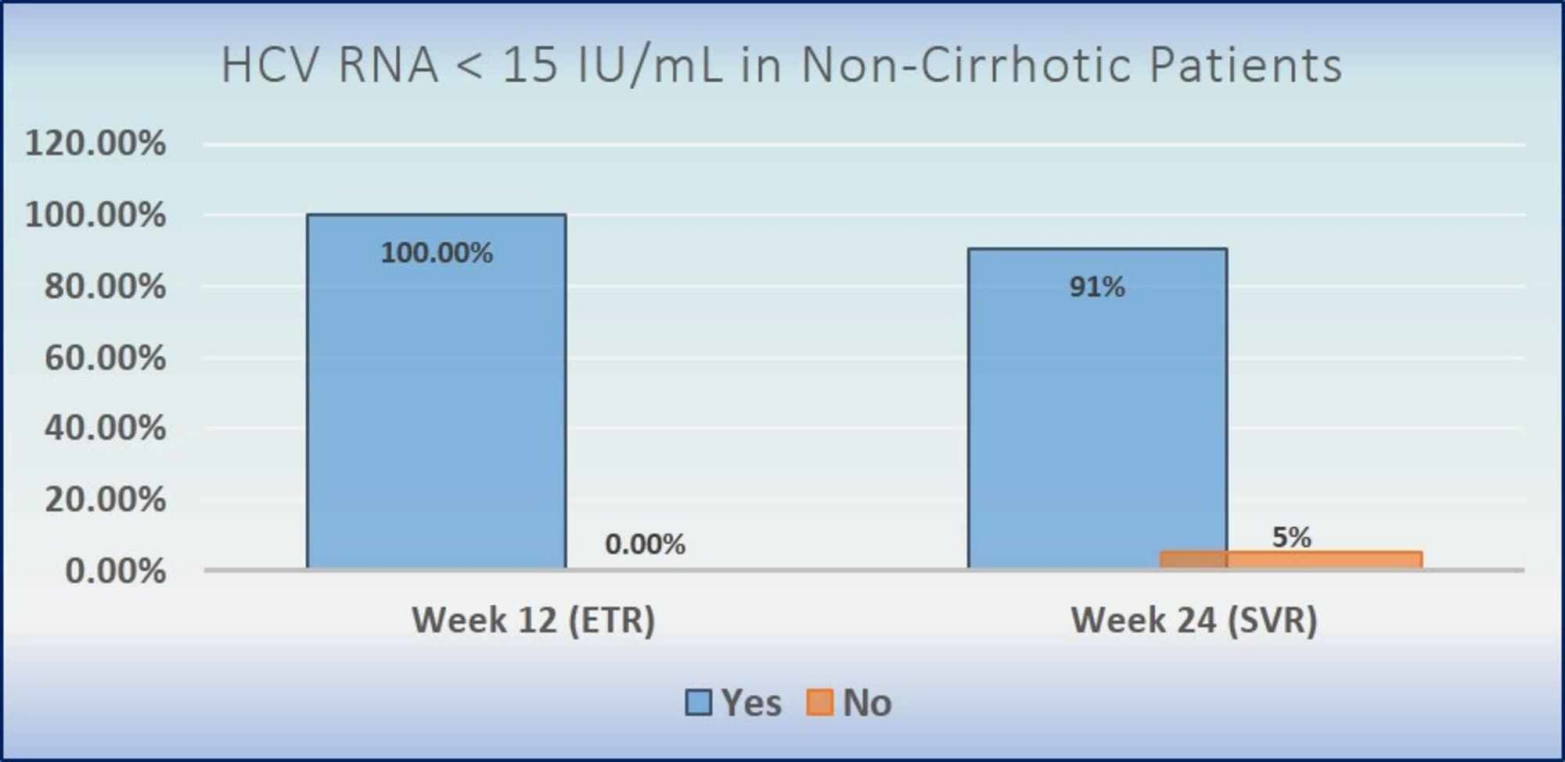
Hepatitis C virus RNA <15 IU/mL achieved in patients without cirrhosis (intention-to-treat population) HCV RNA: hepatitis-C virus ribonucleic acid; ETR: end-of-treatment response; SVR: sustained virologic response

**Figure 3:**
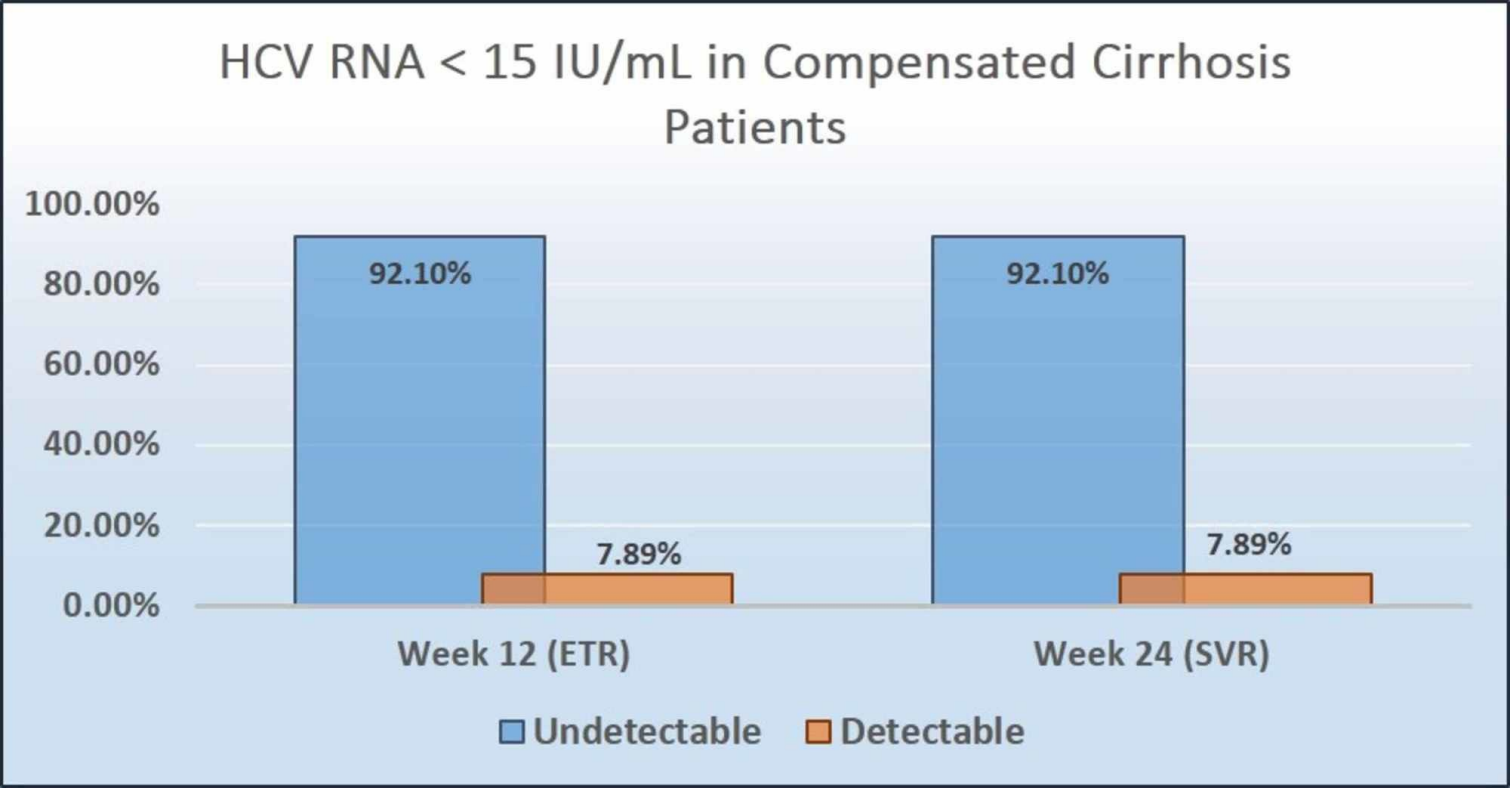
Hepatitis C virus RNA <15 IU/mL achieved in patients with compensated cirrhosis (intention-to-treat population) HCV RNA: hepatitis-C virus ribonucleic acid; ETR: end-of-treatment response; SVR: sustained virologic response

As per the intention-to-treat analysis, all patients without cirrhosis and 35 (92.1%) patients with compensated cirrhosis achieved ETR (undetectable viral load, HCV RNA: <15 IU/mL) at 12 weeks from the start of treatment. A total of 90.5% (95% CI: 84.2-95.8) of patients without cirrhosis and 92.1% (95% CI: 84.2-100) of patients with compensated cirrhosis achieved SVR 12 weeks after the end of therapy (Figure 2, Figure 3). The per-protocol analysis showed 92.5% (95% CI: 87.1-97.8) achievement of SVR among non-cirrhosis hepatitis C patients. Details related to virological response of the sofosbuvir-velpatasvir combination is depicted in Table-2.

**Table 2:** Virologic response of the sofosbuvir-velpatasvir combination (intention-to-treat and per-protocol analysis) RNA: ribonucleic acid; n/N: number of patients/total number of patients

Hepatitis C virus RNA <15 IU/mL achieved after treatment				
	Patients without cirrhosis (n = 95)		Patients with compensated cirrhosis (n = 38)	
Intention-to-treat Analysis	Yes, n/N (%)	No, n/N (%)	Yes, n/N (%)	No, n/N (%)
Week 12 (end-of-treatment response), n = 133	95/95 (100)	0 (0)	35/38 (92.1)	3/38 (7.89)
Week 24 (sustained virologic response), n = 133	86/95 (90.5)	7/95 (5.26)	35/38 (92.1)	3/38 (7.89)
95% CI, week 24	84.2–95.8	4.2–15.8	84–2-100	0–15.8
Per-protocol analysis				
Week 24 (sustained virologic response)	86/93 (92.5)	7/93 (7.5)	35/35 (100)	0
95% CI, week 24	87.1–97.8	2.2–12.9	84.2100	__

Patients with compensated cirrhosis experienced more adverse events (31.5%) than patients without cirrhosis (20.15%). No consistent, clinically signiﬁcant trends were observed when looking at adverse event rates by age group. None of the patients without cirrhosis or with compensated cirrhosis experienced any serious adverse event. A few (two non-cirrhotic and three compensated cirrhotic) patients experienced diarrhea during the study. Certain laboratory abnormalities were seen in both patients without cirrhosis and patients with compensated cirrhosis. All reported adverse events were mild to moderate in severity grading (grade 1 or 2). Specific adverse events and laboratory abnormalities are depicted in Table 3.

**Table 3:** Adverse events and laboratory abnormalities in patients without cirrhosis and in patients with compensated cirrhosis All adverse events reported were of mild to moderate severity. TLC: total leucocyte count; ULN: upper limits of normal

	Patients without cirrhosis (n = 95)	Patients with compensated cirrhosis (n = 38)
Number of patients experiencing any adverse event	20 (21.05%)	12 (31.5%)
Serious adverse event	0	0
Adverse event leading to discontinuation of sofosbuvir-velpatasvir	0	0
Deaths	0	0
Diarrhea	2 (2.1%)	3 (7.89%)
Laboratory abnormalities in either group		
Hemoglobin <10 g/dL	2 (2.1%)	11 (28.9%)
Platelets <90/mm x 10^3^/mm^3^	3 (3.15%)	7 (18.42%)
TLC <4 x 10^9^/L	0	2 (5.2%)
Total bilirubin >2.5 x ULN	0	0

## Discussion

In our study, sofosbuvir-velpatasvir for 12 weeks was found to be highly effective and generally safe and well-tolerated in patients without cirrhosis or with compensated cirrhosis. In Pakistan, as per published meta-analysis, mean HCV prevalence is 6.2% in the general population, 34.5% among the high-risk clinical population, 55.9% among the population with liver-related conditions, and 53.6% among people who inject drugs [15]. Such high prevalence motivated us to undertake this trial to generate real-world clinical data for the treatment of hepatitis C in specific clinical populations in Pakistan.

The nearly identical SVR rates in patients without cirrhosis (92.5%) and patients with compensated cirrhosis (92.1%) support the use of the sofosbuvir-velpatasvir combination for the treatment of hepatitis C in these populations, which is also recommended in the EASL 2018 guidelines [14]. The observed SVR rates in our study are very comparable to the results of the Angioplasty and Stenting for Renal Artery Lesions (ASTRAL)-1 trial that included patients with HCV genotypes 1 to 6, excluding genotype 3, and reported an SVR rate of 99% [16]. ASTRAL-2 and -3 trials reported SVR rates of sofosbuvir-velpatasvir in genotype 2 and 3 patients as 99% and 94%, respectively (also comparable to our study results) [17].

Ten patients did not achieve SVR at 24 weeks from the start of therapy; seven of them were without cirrhosis and three with compensated cirrhosis. Among treatment-failure patients without cirrhosis, two were lost to follow-up after 12 weeks from the start of therapy. Among treatment-failure patients with compensated cirrhosis, all three were unable to achieve ETR at 12 weeks from the start of therapy.

In general, our study results depict a tolerable safety profile for sofosbuvir-velpatasvir among both patients without cirrhosis and patients with compensated cirrhosis, as the majority of adverse events were of mild-to-moderate severity. This interventional trial of sofosbuvir-velpatasvir is the first of its kind in reporting adverse events in the Pakistani population with hepatitis C. The adverse event proﬁle for patients with compensated cirrhosis was consistent with that of advanced liver disease. As interferon-free, direct-acting antiviral-based regimens have only recently become available for the treatment of HCV, the clinical beneﬁts of their use in patients with decompensated cirrhosis are being characterized. Our study results are comparable with the ASTRAL-1 trial in which treatment with the single-tablet regimen of sofosbuvir-velpatasvir for 12 weeks was highly effective for a broad range of patients with HCV. The treatment was also effective among patients with compensated cirrhosis [16]. 

Our study has several limitations, mostly related to the characteristics of the enrolled patients. Primarily, there was a lack of genotype diversity. The study included patients with compensated cirrhosis only (CTP class A). Most of our study patients were treatment-naïve. Lastly, although early improvements in liver function were demonstrated through the post-treatment period during our study, the long-term clinical beneﬁt of achievement of SVR in patients with compensated cirrhosis can only be demonstrated through the follow-up of the patients after the study. Further studies could also be conducted to determine the efficacy and safety of the sofosbuvir-velpatasvir combination in decompensated cirrhosis patients in the local clinical settings. 

## Conclusions

HCV infection is endemic in Pakistan and some other developing countries, and its burden is expected to increase in the coming years. Our study showed that a full daily dose of the sofosbuvir-velpatasvir combination for 12 weeks is safe and efficacious in hepatitis C patients without cirrhosis or with compensated cirrhosis, irrespective of the genotype.
